# Microbiome and cancer: from mechanistic implications in disease progression and treatment to development of novel antitumoral strategies

**DOI:** 10.3389/fimmu.2024.1373504

**Published:** 2024-04-23

**Authors:** Marian Constantin, Mariana Carmen Chifiriuc, Grigore Mihaescu, Nicolae Corcionivoschi, Liliana Burlibasa, Coralia Bleotu, Sorin Tudorache, Magda Mihaela Mitache, Roxana Filip, Simona-Gloria Munteanu, Gratiela Gradisteanu Pircalabioru

**Affiliations:** ^1^ Institute of Biology, Bucharest of Romanian Academy, Bucharest, Romania; ^2^ Life, Environmental and Earth Sciences Division, Research Institute of the University of Bucharest, Bucharest, Romania; ^3^ Faculty of Biology, University of Bucharest, Bucharest, Romania; ^4^ Bacteriology Branch, Veterinary Sciences Division, Agri-Food and Biosciences Institute, Belfast, United Kingdom; ^5^ Faculty of Bioengineering of Animal Resources, Banat University of Agricultural Sciences and Veterinary Medicine-King Michael I of Romania, Timisoara, Romania; ^6^ Romanian Academy of Scientists, Bucharest, Romania; ^7^ Stefan S. Nicolau Institute of Virology, Bucharest, Romania; ^8^ Faculty of Medicine, Titu Maiorescu University, Bucharest, Romania; ^9^ Faculty of Medicine and Biological Sciences, Stefan cel Mare University of Suceava, Suceava, Romania; ^10^ Suceava Emergency County Hospital, Suceava, Romania; ^11^ Carol Davila University of Medicine and Pharmacy, Bucharest, Romania; ^12^ eBio-Hub Research Centre, National University of Science and Technology Politehnica Bucharest, Bucharest, Romania

**Keywords:** cancer, microbiota, microbiome, immunotherapy, probiotics

## Abstract

Cancer is a very aggressive disease and one of mankind’s most important health problems, causing numerous deaths each year. Its etiology is complex, including genetic, gender-related, infectious diseases, dysbiosis, immunological imbalances, lifestyle, including dietary factors, pollution etc. Cancer patients also become immunosuppressed, frequently as side effects of chemotherapy and radiotherapy, and prone to infections, which further promote the proliferation of tumor cells. In recent decades, the role and importance of the microbiota in cancer has become a hot spot in human biology research, bringing together oncology and human microbiology. In addition to their roles in the etiology of different cancers, microorganisms interact with tumor cells and may be involved in modulating their response to treatment and in the toxicity of anti-tumor therapies. In this review, we present an update on the roles of microbiota in cancer with a focus on interference with anticancer treatments and anticancer potential.

## 1 Introduction

The etiology of cancer is characterized by multiple factors, encompassing: (1) genetic influences, involving hereditary elements linked to inherited genetic predispositions, such as specific mutations, locus weakening, deletions, activation of proto-oncogenes, and inactivation of tumor suppressor genes; (2) external factors, encompassing exposure to carcinogens or procarcinogens; (3) internal factors, associated with the metabolism of procarcinogens into carcinogens, modifications in the local microenvironment, stress conditions leading to the production of reactive oxygen species, and compromised or abnormal functioning of the immune system; and (4) microbial factors, particularly in instances where the microbiota undergoes alterations (dysbiosis) and becomes enriched with microorganisms producing genotoxic compounds.

The gut microbiome has been recognized as an important player in cancer development and progression (1).

Microbiota, comprising bacteria, fungi, microalgae, or protozoa, along with viruses, have the potential to instigate or advance neoplastic processes. This can occur through the direct synthesis of carcinogenic compounds, exerting genotoxic effects, direct inactivation of genes, initiation of local inflammatory processes leading to alterations in the local microenvironment, or impairment of the immune system’s functionality, resulting in immunosuppression. Recent developments also indicate that the microbiome holds an important role in modulating the efficacy and toxicity of chemotherapeutic drugs (irinotecan, 5-fluorouracil, oxaliplatin, cyclophosphamide, gemcitabine, methotrexate) and immunotherapeutic compounds (anti-cytotoxic T-lymphocyte-associated antigen 4, anti-programmed death-ligand 1/anti-programmed cell death protein 1) (2, 3). Oncologic outcomes are highly impacted by the microbiome composition and functions, hence there is an imperative need to develop personalized treatment approaches targeting the microbiota.

Within this line of thought, this review aims to present the influence of the gut microbiota in antitumoral therapies, and the ways of modulating the microbiome in cancer treatment.

### 1.1 Positive influence of microorganisms on antitumor therapies

Commensal and symbiotic bacteria colonize numerous surfaces in the host organism and are also found in sites originally considered sterile, such as the placenta, blood, breast milk and tumors. Therefore, the use of bacteria as therapeutic agents for various tumors is an attractive area of study. The first findings on the role of bacteria in cancer therapy date back to 1813, when the French physician Arsène-Hippolyte Vautier observed the apparent healing of tumors in people with gas gangrene produced by *Clostridium perfringens* (4). In 1868, the German physician Karl David Wilhelm Busch infected a cancer patient with erysipelas, achieving rapid tumor shrinkage, but the patient died 9 days from infection. In 1883, the German surgeon Friedrich Fehleisen identified *Streptococcus pyogenes* as the causative agent of erysipelas and confirmed that it could reduce the size of tumors. Similarly, in 1891, the American surgeon William Bradley Coley confirmed that severe *Streptococcus pyogenes* infection can cure people with cancer. Although he was an orthopedic surgeon, he dedicated his life to researching tumor reduction with bacterial extracts from inactivated streptococci and *Serratia marcescens*, discovering the so-called ‘Coley’s toxin’, hypothesizing that tumor destruction occurs through an immune reaction induced by the bacterial preparations, thus he is considered the founder of cancer immunotherapy (5). Because the effectiveness of William Bradley Coley’s treatment depended on several factors, many of them related to his ability to determine the immune response conducive to tumor destruction, with the entry of radiotherapy and chemotherapy into standard cancer therapies, ‘Coley’s toxin’ was left aside (4). Towards the end of the 19^th^ century and the beginning of the 20^th^ century, a reduced frequency of cancer cases was observed among people with tuberculosis, the link between the two pathologies being elucidated only after the isolation, in 1921, of the bacillus Calmette-Guérin, which represents an attenuated strain of *Mycobacterium bovis* used as an antituberculosis vaccine. The Calmette-Guérin vaccine is used for immunotherapy of advanced bladder cancer (6). We now know that bacteria colonize tumors and the mycobacterial antigens are triggering an immune response active against both bacteria and infected tumor cells. There are also bacteria that secrete toxins contributing to the lysis of eukaryotic cells, and finally, genetically engineered bacteria that can deliver, targeted to the tumor, different cytokines that cause immune suppression of neoplastic cells. Some microorganisms are directly or indirectly involved in the neoplastic process, while others colonize solid tumors, the most well-known being *Bifidobacterium longum*, *Bifidobacterium adolescentis*, *Clostridium histolyticus*, *Clostridium butyricum*, *Clostridium novyi*, *Clostridium beijerincki*, *Escherichia coli*, *Listeria monocytogenes*, *Salmonella choleraesuis*, *Salmonella enterica* serovar *typhimurium*, *Salmonella typhimurium* and *Pseudomonas aeruginosa*. In addition to bacteria, the use of viruses in anti-tumor treatment appears to be showing some results.

By integrating the multidisciplinary knowledge and experience, the field of pharmacomicrobiomics has been proposed aiming to investigate the complex relationships between microbiota and drugs and to identify ways to use commensal, probiotic, prebiotic, postbiotic and symbiotic microorganisms or those in fecal matter for the development of personalized therapies that offer better chances of cure (**Table 1**).

**Table 1:** Potential of using microorganisms in curative cancer treatment.

Microorganism	Role	Discoverer	References
*Clostridium perfringens*	Apparent healing of tumors	Arsène-Hippolyte Vautier, 1813	Rius-Rocabert, 2019 (4)
*Streptococcus pyogenes*	Decrease of tumors and curing cancer patients	Friedrich Fehleisen, 1883 William Bradley Coley, 1891	Van Mellaert, 2006 (5)
*Mycobacterium bovis* (Calmette-Guérin vaccine)	Stimulation of antitumor cellular response in advanced bladder cancer	Léon Charles Albert Calmette, Jean-Marie Camille Guérin, 1921	Sylvester, 2002 (6)
*Salmonella typhimurium* ΔppGpp	Activation of inflammasome signaling pathways		Phan, 2015 (7)
*Escherichia coli* K-12 strain (MG1655)	Proliferation of T lymphocytes		Phan, 2015 (7)
*Salmonella enterica* serovar *typhimurium*	Stimulation of TNFA production		Phan, 2015 (7)
*Pseudomonas aeruginosa*	Inhibition of metastasis formation		Chang, 2015 (8)
*Helicobacter pylori*	Stimulation of NK cells to produce IFN-γ		Yun, 2005 (9)
*Clostridium novyi*	Decrease of tumor volume by producing lytic toxins		Roberts, 2014 (10)

### 1.2 Stimulation of the antitumoral immune response by microbial products

Commensal and symbiotic microbiota play an important role in the education and maturation of the immune system in human (11), having thus an important role in oncogenesis, tumor progression, and in response to treatment, through mechanisms that are involving the immune system and its relationship with the tumor microenvironment, forming the so-called tumor immune microenvironment (12).

**Figure 1:**
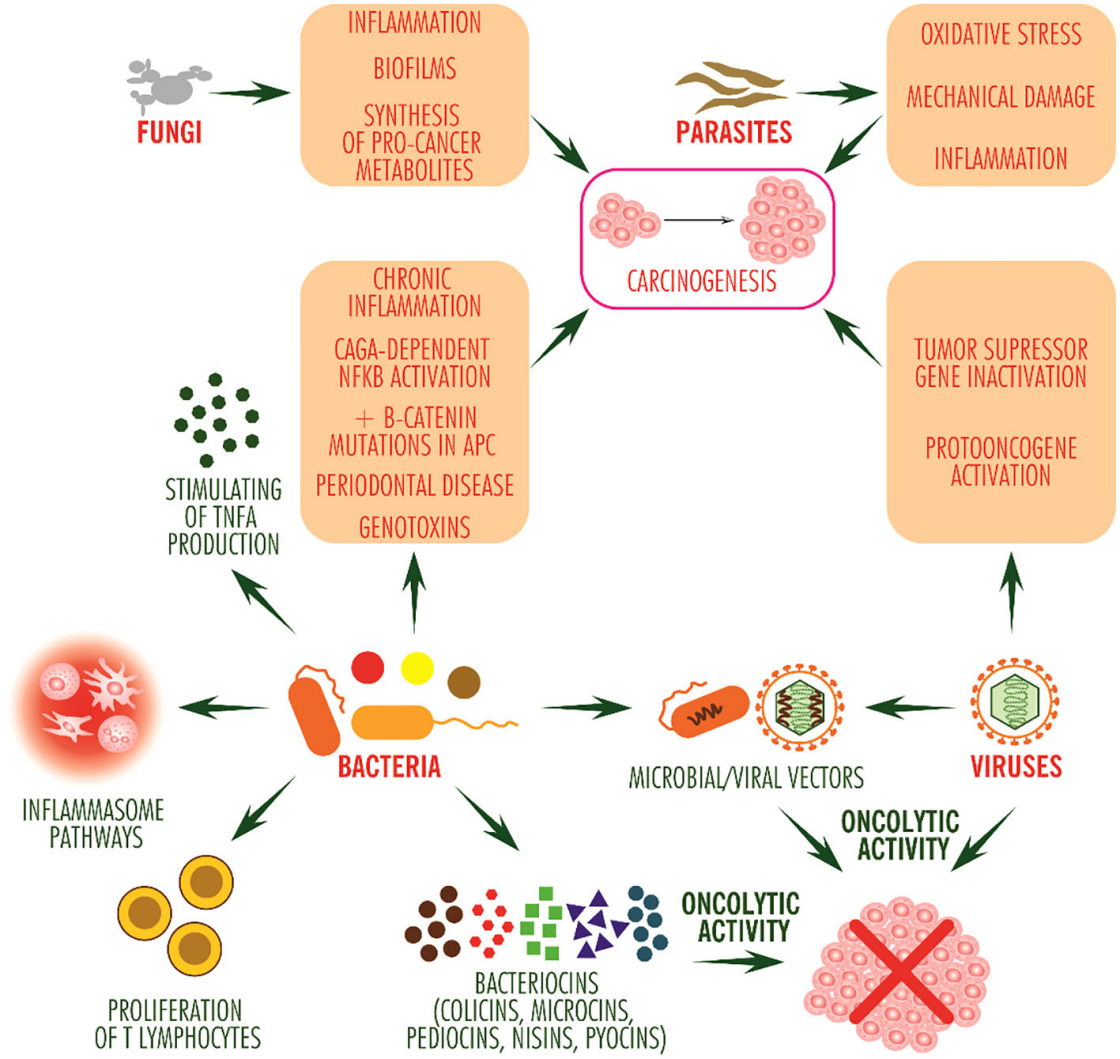
Pro- and anti-tumor potential of microorganisms. The anti-tumor effect of bacteria occurs through stimulating inflammasome signaling pathways and by stimulating T lymphocyte proliferation and TNF alpha production, and by bacteriocins, mainly directed against other bacterial competitors, may exhibit oncolytic activity. Viruses stimulate carcinogenesis, with some having oncolytic activity (such as picornavirus ECHO-7). Bacteria and viruses can be used as vectors for the delivery of antitumor molecules. Fungi and animal parasites mainly induce inflammation. In addition, fungi contribute to the formation of bacterial biofilms, colonize tissues with bacteria and protect them against the immune system while synthesizing pro-tumor metabolites such as acetaldehyde, and parasites induce inflammation due to oxidative stress and mechanical damage to tissues.

Although the number of bacterial species that can colonize solid tumors is generous, only a few species, *Salmonella typhimurium/Salmonella enterica* serovar *typhimurium*, *Escherichia coli*, *Pseudomonas aeruginosa*, *Listeria monocytogenes*, *Clostridium sporogenes* and *Clostridium novyi* (13), play an effective role in immune shaping of the tumor microenvironment (**Figure 1**).

The tumor microenvironment (tumor stroma) is a complex entity comprising a highly diverse cellular component consisting of genetically transformed stromal cells including endothelial cells, adipocytes, cancer-associated fibroblasts, peripheral nervous system-derived nerve fibers, blood or lymphatic cells, infiltrating immune nonspecific and specific cells (macrophages, neutrophils, antigen-presenting dendritic cells and myeloid-derived suppressor cells, NK cells, T lymphocytes and B lymphocytes), neuroendocrine cells, an extracellular matrix component, comprising collagen, laminin, fibronectin, elastin and tenascin, and a whole range of cytokines, such as CSF1 (Colony Stimulating Factor 1), chemokines, such as CCL1-5 and 7 (C-C Motif Chemokine Ligand 1-5 and 7), CXCL1-2 (C-X-C Motif Chemokine Ligand 1-2), CXCL4 (C-X-C Motif Chemokine Ligand 4) and CX3CL1 (C-X3-C Motif Chemokine Ligand 1), and by growth factors secreted by any of the cells present in this environment (14, 15). The interactions among the components of the tumor microenvironment lead to reprogramming of the immune response, either towards stimulation or inhibition of tumor progression (14), this duality of the immune system being one of the important factors in spontaneous tumor regression (16). In addition to the tumor microenvironment, immune reprogramming is influenced to some extent by the microbiota colonizing the tumor and the body, which could act as silent colonizers or as pathogens, and, under certain conditions, they could reduce or eliminate the tumor being regarded as potential anti-tumor agents (12). Microorganisms that colonize tumors can compete with tumor cells for nutrients in the tumor microenvironment, starving them and slowing their expansion can activate inflammasome signaling pathways, affecting the signals emitted by tumor cells and contributing to the strengthening of the immune system (17), which can fight tumors more effectively as well, and can damage tumor cells, inducing apoptosis. The inflammasome is a major class of signalosomes in innate immunity and comprises a broad range of cytosolic proteins that produce inflammation and direct cells to pyroptosis, the programmed cell death due to inflammation. The pyroptotic inflammasome is frequently encountered in tumorigenesis and is associated with many cancers (18). Intravenous injection of modified and attenuated strain of *Salmonella typhimurium*, defective in ppGpp synthesis (ΔppGpp) (7), a facultatively anaerobic bacteria that can colonize normoxic and hypoxic tissues, results in its intratumoral accumulation and inflammasome activation (19), with abundant secretion of the inflammatory cytokines IL1 beta, secreted after activation of TLR4 (Toll like receptor 4) with lipopolysaccharide, IL18 and TNF alpha, which suppress tumor growth. *Salmonella typhimurium* ΔppGpp can activate the inflammasome in bone marrow derived macrophages, which produce tumor cell injuries, the damaged cells being then phagocyted by macrophages (7). Shortly after inoculation, *Salmonella enterica* serovar *typhimurium* colonizes tumors, inducing rapid increases in proinflammatory cytokine and TNF alpha levels, the latter producing endothelial cell injury. These lead to vascular ruptures within the tumor, intratumoral hemorrhages and necrosis of tumor tissue, which favor the accumulation of neutrophils in the necrotic focus to separate it from healthy tissue. Outnumbering neutrophils, bacteria escape from the necrotic focus into viable tumor cells, multiplying, intensely colonizing the tumor and extending TNF-α synthesis and necrosis (20). Thus, *Salmonella enterica* serovar *typhimurium* contributes to significant shrinkage for 7-10 days after inoculation. The effect is only temporary, as subsequently the tumor resumes its proliferation tendency (7).

Inoculation with *Escherichia coli* K-12 strain (MG1655), which exhibits tumoral accumulation tendency and contributes to clearance of some tumor cell types, such as the murine colon carcinoma line CT26, stimulates T lymphocyte activation. Of these, CD8+ T lymphocytes exhibit cytotoxic activity on tumor cells, and CD4+ and CD8+ lymphocytes suppress tumor recurrence and achieve tumor cell clearance, in the memory phase (21). CD4+ and CD8+ regulatory T lymphocytes contribute to the reduction of colon inflammation and may intervene in the prevention of colon cancer (19). Along with *Bacteroides thetaiotaomicron* and *Bacteroides caccae*, *Bacteroides fragilis* enhances dendritic cell activity through TLR4 expression and IL12 synthesis, which further activates T helper lymphocytes, which secrete IFN-γ, with antitumor effect (22, 23). In people with metastatic melanoma, *Faecalibacterium prausnitzii* contributes to the stimulation of CD8+ T lymphocyte cytotoxic activity and the increase of CD4+ and ICOS+ (Inducible T-cell costimulatory) T lymphocytes, in parallel with the amelioration of colitis and the reduction of regulatory T lymphocytes and myeloid-derived suppressor cells, which have a positive role in tumorigenesis (24). Oral administration of mixtures of *Bacteroides fragilis* and *Bacteroides thetaiotaomicron* or *Bacteroides fragilis* and *Burkholderia cepacia* to mice with tumors and whose gut microbiota had previously been ablated resulted in reduced tumor volume (25). *Bifidobacterium* species also activate cytotoxic T lymphocytes, *Bifidobacterium longum*, *Bifidobacterium breve* and *Bifidobacterium pseudolongum *via MHC II (Major histocompatibility complex II)/HLA-DM (HLA Class II Histocompatibility Antigen, DM) molecules, HLA-DO (HLA Class II Histocompatibility Antigen, DO), HLA-DP (HLA Class II Histocompatibility Antigen, DP), HLA-DQ (HLA Class II Histocompatibility Antigen, DQ) and HLA-DR (HLA Class II Histocompatibility Antigen, DR) on dendritic cells and *Bifidobacterium bifidum* via peptidoglycans.

Preparations of *Pseudomonas aeruginosa* can help inhibit metastasis formation and improve immune system function in people undergoing chemotherapy treatment without significant toxic effects (8). Other studies have attempted to use *Helicobacter pylori* to induce an anti-tumor immune response in the presence of low levels of IL12 produced by macrophages and dendritic cells, but their efficacy is questionable. Initially, *Helicobacter pylori* antigens were shown to induce NK cells in the stomach and duodenum to synthesize IFN gamma (9), but without therapeutic applicability as this does not occur in gastric cancer patients (26). Inoculated intratumorally as spores, the anaerobic bacterium *Clostridium novyi* infects hypoxic areas of tumors (27), producing infection-specific symptoms (fever and pain), and a strong immune response, tumor cell death and reducing tumor volume.

Bacteria can be engineered to deliver human genes into infected tissues, which they express locally and induce an anti-tumor immune response. Some of the most widely used bacterial vectors are derived from *Salmonella typhimurium*, which can be modified to secrete LIGHT/TNFSF14, IL18, FASL and TRAIL cytokines, and when injected intravenously into laboratory animals, contribute to the tumor reduction *in vivo*. Thus, LIGHT/TNFSF14 (Tumor necrosis factor ligand superfamily member 14) is involved in the infiltration of B lymphocytes and CD4+ and CD8+ T lymphocytes into the tumor microenvironment (28), IL18 promotes leukocyte influx, especially of NK cells, into the tumor microenvironment, and abundant secretion of INF gamma, TNF alpha, IL1 beta and GM-CSF (granulocyte-macrophage colony-stimulating factor) (29), FASL contributes to neutrophil recruitment (30), and TRAIL (TNF-related apoptosis-inducing ligand), induces caspase 3 and 8-dependent apoptosis (31), all of which promote tumor cell recognition and destruction. Also, in preclinically tested animal models, attenuated *Salmonella typhimurium* genetically modified to produce CCL21 induces intratumoral increases in IFN-γ, CXCL9 and CXCL10 levels and tumor reduction in a CD4+ and CD8+ expressing lymphocyte-dependent manner, providing safety in use (32). The use of *Listeria monocytogenes* containing ADXS31-164 HER-2/neu induced increased TCD8+/Tregs ratio in the tumor microenvironment, preventing primary tumor growth and delaying metastasis (33). *Clostridium* species can be engineered to deliver molecules to tumors that decrease their aggressiveness without producing negative effects on the body. Of these, *Clostridium novyi*-NT AC and *Clostridium sporogenes* NCIMB 10696 are modified to produce anti-HIF1A antibodies, inhibiting the angiogenic signaling pathway at an early stage (34), and *Clostridium sporogenes* ATCC 3584 is modified to deliver IL12, selectively amplifying IFN gamma secretion and affecting tumor growth (35).

### 1.3 Oncolytic bacteria

In competition for nutrients, bacteria secrete various compounds to inhibit the growth of the competitors, some of which are also inhibiting the tumoral growth. Bacteriocins and some bacterial metabolites, including phenazine metabolites, are useful in this regard. Also, bacteria can be engineered to produce different metabolites of interest.

Bacteriocins are positively charged, cationic proteins secreted by most bacterial species. They are non-immunogenic and biodegradable, bypass the electrically neutral membranes of healthy cells and become selectively attached to the negatively charged membranes of tumor cells (36). Among the bacteriocins that can be used in the treatment of cancers are colicins, microcins, pediocins, nisins and pyocins. Colicins are bacteriocins produced by *Escherichia coli* and other bacterial species of the *Enterobacteriaceae* family and affect the proliferation of a large number of tumor cell lines, including colon, breast, bone and HeLa cells (37). Microcin E492, produced by *Klebsiella pneumoniae* RYC492, which forms pores in eukaryotic cell membranes, can induce apoptosis in some human cells, including colorectal carcinoma cells, HeLa cells, Jurkat cells (acute T-cell leukemia-derived cell line) and RJ2.2.5 cells (cell line derived from a variant of Burkitt’s lymphoma), without affecting KG-1 cells (lymphoblast-like cells derived from the bone marrow of a patient with acute myelogenous leukemia) or human tonsil endothelial cells in primary culture (38). Nisin is a bacteriocin produced by several *Lactobacillus* and *Streptococcus* species during fermentation, is used as a preservative, is non-toxic and safe for use in humans, and induces apoptosis of HepG2 cell lines (hepatocellular carcinoma-derived cells) (39), MCF-7 (human mammary adenocarcinoma-derived cells), HT-29 (human colon cancer-derived cells) (40), on HUVEC (human umbilical vein endothelial cells) and on head and neck squamous cell carcinoma-derived cells (41). Pyocins are bacteriocins produced by the vast majority of *Pseudomonas aeruginosa* individuals, one of which destroys mouse fibroblast-derived L6OT cells (42), while pyocin S2, secreted by *Pseudomonas aeruginosa* 42A, exhibits cytolytic activity on the tumor cell lines HepG2 and Im9 (human multiple myeloma-derived immunoglobulin-secreting cell line), with no effect on the normal cell line HFFF (Human fetal foreskin fibroblast) (43). Besides bacteriocins, other metabolites such as phenazine metabolites, secreted by strains of *Pseudomonas aeruginosa* and other bacterial species, such as *Streptomyces* sp., can exhibit cytotoxic activity on tumor cell lines. Thus, phenazine 1,6-di-carboxylic acid strongly affects the viability of HeLa, HT29 and MCF-7 cell lines and, to a lesser extent, that of the DU145 line (derived from human prostate cancer) (44).

### 1.4 Oncolytic viruses and viral vectors

Unlike oncolytic bacteria, which produce different metabolites, oncolytic viruses indirectly activate systemic antitumor immunity and cause lysis of tumor cells, which they selectively infect (45). Among the viruses used or tested for use in cancer treatment are viruses with a DNA genome, including HSV1 (herpes simplex virus type 1), vaccinia virus and some adenoviruses, and viruses with an RNA genome, including reoviruses, measles virus and VSV (vesicular stomatitis virus) (46), to which genes for cytokines, checkpoint inhibitors and antigenic or immunostimulatory molecules have been inserted (47). Among the modified or attenuated oncolytic adenoviruses, ONYX-015, with deletion of the 55kD E1B gene (48), OBP-301 (Telomelysin), with insertion of the promoter for hTERT (human telomerase reverse transcriptase), are being tested for replication in tumor cells, which overexpress hTERT (49), VCN-01, with tropism for RB-deficient tumor cells (Retinoblastoma) (50), and CAdVEC, a binary vector, derived from an oncolytic adenovirus and a helper-dependent adenovirus and with integrated genes for IL12 and PDL1 (CAdVECIL12_PDL1) and HER2 (human epidermal growth factor receptor 2) (51). Among the herpesvirus-based vectors tested are HF10, which can induce tumor necrosis in a CD8+ T lymphocyte-dependent manner and the release of several cytokines including IL2, IL12, TNF alfa, IFN alpha, IFN beta and IFN gamma (52), and ONCR-177, derived from HSV1, containing genes for IL12, CCL4, for PD1 and CTLA4 antagonists and the sequence for the extracellular domain of FLT3LG and inducing T lymphocyte and NK cell activation, and dendritic cell expansion and recruitment (53). Other viruses tested in antitumor therapy are: MV-NIS, cavatak (Coxsackievirus A21 or CVA21), Reolysin (Pelareorep), GL-ONC1 (GLV-1h68), and JX-594 (Pexa-Vec), while T-VEC (thalimogene laherparepvec), derived from HSV1, Rigvir (Riga virus), an unmodified picornavirus ECHO-7 (Enteric Cytopathogenic Human Orphan type 7), and Oncorine (H101), a modified adenovirus, are effectively used in the treatment of some cancers (54).

## 2 Synergistic effect of microbiota and anticancer therapy

The gut microbiota has long been known to play an important role in maintaining health and triggering disease, with increasing evidence recently also accumulating on its positive involvement in modulating the effectiveness of chemotherapy and immunotherapy (55). On the other hand, there are studies showing that ablation of the gut microbiota by antibiotics reduces the efficacy of some cancer treatments (56). The mechanisms by which the microbiota promotes positive response to treatment are not fully known, but various studies in the field suggest that they include epigenetic regulation of gene expression, modulation of immune response, enzymatic degradation and drug metabolism (55).

Administered in low doses, cyclophosphamide (CTX), an alkylating agent with antiangiogenic and immunostimulatory effects, causes translocation of Gram-positive intestinal bacteria, such as *Enterococcus hirae*, to secondary lymphoid organs, stimulating intratumorally increase in the TCD8+/TCD4+ ratio (56) and differentiation of CD4+ T helper 17 (Th17) lymphocytes. Th17 lymphocytes induce an inflammatory reaction in tumor tissues that increases the tumoricidal effect of CTX (57). On the other hand, during CTX treatment, *Barnesiella intestinihominis* accumulates in the colon and promotes infiltration of Tγδ lymphocytes into tumor lesions, where they produce IFN gamma (56). These effects do not occur in germ-free mice, which easily acquire resistance to CTX (57).

CpG oligonucleotides are used in cancer immunotherapy. They form a complex with the TLR9 receptor (Toll-like receptor 9) and activate macrophages, monocytes, dendritic cells, NK cells and B and T lymphocytes, fostering the synthesis of INF alpha (58). The intestinal microorganisms, in particular *Ruminococcus* sp. and *Alistipes shahii*, are positively correlated with TNF and granzyme B secretion and play an important role in triggering an antitumor immune response (23).

Oxaliplatin is a new-generation chemotherapeutic agent used in the first-line treatment of many cancers, including skin and advanced cancers of the appendix, colon and rectum. Its mechanism of action is based on the delivery of platinum ions into the nuclei of tumor cells, where they form adducts with genetic material, block replication and cause cell death (59). Oxaliplatin has lower toxicity and higher efficacy than previous similar drugs (cisplatin, carboplatin) and, unlike them, induces immunogenic cell death and promotes anti-tumor T-lymphocyte immunity. Administered to laboratory animals with intact gut microbiota, oxaliplatin destroys subcutaneous EL4 tumors and prolongs survival, an activity greatly diminished in previously antibiotic-treated animals. Finally, germ-free animals did not respond to oxaliplatin treatment (60). Among the microorganisms that contribute to oxaliplatin efficacy are *Bacteroides fragilis*, which stimulates infiltration of CD8+ cytotoxic T lymphocytes into the tumor microenvironment (61), and microorganisms of the genera *Ruminococcus*, *Clostridium* (*Clostridium butyricum*), *Eubacterium* (*Eubacterium hallii* and *Eubacterium rectale*), *Coprococcus*, *Faecalibacterium*, *Butyrivibrio* etc., which produce butyric acid (62). This is a short-chain fatty acid that, in low concentration, enhances the activity of tumor-infiltrating CD8+ cytotoxic T lymphocytes and, in synergy with the probiotic *Bifidobacterium bifidum*, causes increased production of granzyme B (63).

Fluoropyrimidines are fluorinated derivatives of uracil and are used as antimetabolites in the treatment of several types of cancers to induce DNA breaks, disruption of DNA replication and RNA synthesis, and ultimately cell death (64, 65). These drugs, such as intravenously administered *5-*fluorouracil (5-FU) and orally administered capecitabine, are predominantly converted in tumor cells and liver to the cytotoxic 5-fluorouracil form of thymidine phosphorylase. In the presence of 5-fluorouracil, *Lactobacillus acidophilus* CL1285 and *Lactobacillus casei* LBC80R strains induce apoptosis of LS513 human colorectal cancer cells (66), and in HER2-negative metastatic breast cancer, the abundance of microorganisms such as *Slackia* and *Blautia obeum* is associated with progression-free survival (67).

CTLA4 inhibitors are a group of drugs designed to block CTLA4 (cytotoxic T-lymphocyte-associated protein 4)/CD152 (cluster of differentiation 152) activity. This receptor is expressed on the surface of regulatory T lymphocytes and, by binding to B7.1 (CD80) protein expressed by cytotoxic T lymphocytes, attenuates their activity (22). Therapeutic blockade of CTLA4 is achieved with immune inhibitors of CTLA4, such as the antibody ipilimumab, which can restore anti-tumor activity to cytotoxic T lymphocytes. Administration of ipilimumab induces intestinal dysbiosis, marked by multiplication of bacteria of the *Clostridiales* genera and reduction of those of the *Bacteroidales* and *Burkholderiales genera*, while the colony density of *Bacteroides fragilis* appears to be unaffected by it (22). Moreover, the high density of *Bacteroides fragilis* stimulated by ipilimumab (23, 25)] is associated with reduced tumor volume. *Enterococcus faecium* induces synergistic antitumor effects of CTLA4 inhibitors via peptidoglycan fragments, which activate CX3CR1+ monocytes, stimulate cytotoxic lymphocytes to produce granzyme B, and reduce the number of tumor-associated macrophages (23).

PD1 (Programmed cell death 1) and PDL1 (Programmed cell death ligand 1) inhibitors, are immunosuppressors of T lymphocytes, impair their function and promote tumor proliferation and invasiveness (68), and their antagonists are used in the treatment of cancers, including the anti-PD1-directed monoclonal antibody products nivolumab, pembrolizumab and durvalumab, and the anti-PDL1-directed atezolizumab and avelumab (69), which specifically target and destroy tumor cells. The presence of some bacterial species in the gut of people with cancer may amplify the immune response of PD1- and PDL1-directed products and prolong their survival. Thus, *Bifidobacterium bifidum*, *Bifidobacterium longum*, *Bifidobacterium breve*, *Bifidobacterium pseudolongum*, *Akkermansia muciniphila* ATCC BAA-835 and *Faecalibacterium prausnitzii* enhance the activity of anti-PD1 antibodies (22, 23). *Enterococcus faecium* has synergistic action with PD1 inhibitors and PDL1 inhibitors via peptidoglycan fragments (23), and *Faecalibacterium* sp. appears to be associated with anti-PDL1 response (70).

The presence of *Escherichia coli*, *Listeria welshimeri*, *Bifidobacterium breve*, *Lactococcus lactis* and *Lactobacillus* sp. in the gastrointestinal tract of people with lung or prostate carcinomas has a synergistic effect with some antitumor drugs. Thus, *Escherichia coli* is associated with enhanced activity of tretazicar (CB1954), fludarabine phosphate, 5-fluorocytosine, 6-mercaptopurine-2-deoxyriboside and gemcitabine, *Listeria welshimeri* with that of fludarabine phosphate and tretazicar (CB1954) (71), *Bifidobacterium breve* and *Lactococcus lactis* with that of tretazicar (CB1954), and *Lactobacillus* sp., with that of 5-fluorocytosine (72).

## 3 Use of probiotics, prebiotics, synbiotics and postbiotics as adjuvants in cancer treatment

Probiotics are cultures of living microbial cells that colonize and benefit the human body. They naturally define the state of eubiosis and can be affected by antibiotic use, different diets and infections with different pathogens. Among the diets that favor their development are those containing prebiotics, oligosaccharides or other non-digestible, fermentable compounds that modify the structure and/or activity of the gut microbiota, with beneficial effects on health. Of these, only fructo-oligosaccharides and galacto-oligosaccharides are generally considered safe, while a wider range of prebiotics, including pectin oligosaccharides, xylo-oligosaccharides, isomalto-oligosaccharides, gluco-oligosaccharides, mannan-oligosaccharides, gentio-oligosaccharides, soybean oligosaccharides, chito-oligosaccharides and polydextrose are still being evaluated for safety, and the number of prebiotics that will become safe may increase in the future (73).

Gut colonization with probiotics, including bacteria *Bifidobacterium infantis*, *Lactobacillus acidophilus*, *Enterococcus faecalis* and *Bacillus cereus*, inhibits the growth of pathogenic microorganisms, including *Desulfovibrio*, *Mucispirillum* and *Odoribacter*, helping to reduce the risk of colon cancer and associated colitis, and the probiotic yeast *Saccharomyces boulardii* inhibits *Bacillota*, *Proteobacteria* and *Tenericutes*, promotes the growth of *Bacteroidetes*, with metabolic modification, and is indicated in the treatment of obesity and type 2 diabetes (74). Probiotics regulate intestinal transit, restore and maintain gut microbiota after antibiotic treatment, reduce intestinal inflammation, secrete butyric acid and propionic acid (short-chain fatty acids) and may contribute to the reduction of tumor volume and number/volume of metastases, acting synergistically with anti-tumor drugs (75, 76). In murine melanoma, short-chain fatty acids synthesized in the intestine and transported via the bloodstream stimulate CCL20 (C-C Motif Chemokine Ligand 20) expression in endothelial cells in lung metastases, recruitment of Th17 lymphocytes, and reduction in the number of lung metastases (77). In experiments on human intestinal Caco-2 cells whose tight junctions were disrupted to reconstitute conditions in small intestinal necrotizing colitis, the probiotics *Lactobacillus rhamnosus* and *Lactobacillus plantarum* restored these junctions, with the cluster of cells regaining intestinal barrier function (78). In murine breast cancer, administration of milk fermented with *Lactobacillus casei* CRL431 reduces the secretion of the pro-angiogenic cytokine IL6, and similarly *Lactobacillus reuteri* isolated from human milk increases the proportion of CD8+/CD4+ T lymphocytes, delaying tumor development (79). Multiple observations on the beneficial effects of probiotics indicate the use of probiotic bacterial strains, especially from the genera *Lactobacillus*, *Bifidobacterium*, *Lactococcus*, *Streptococcus*, *Enterococcus*, and, less so, from the genera *Bacillus* and *Saccharomyces*, for colonization of the gut after antibiotic treatment, which unbalances the gut microbiota.

Furthermore, a diet enriched in *Lactobacillus acidophilus*, such as some cheeses, some kefir, kombucha, is correlated with reduced risk of colorectal cancer incidence in mice. *Lactobacillus rhamnosus* GG strain has proapoptotic and antiproliferative effect on murine (HGC-27) and human (Caco-2, DLD-1 and HT-29) colon carcinoma cells, reducing IL8 levels, *Bacillus polyfermenticus species*, *Bacillus subtilis*, *Bifidobacterium lactis*, *Bifidobacterium adolescentis*, *Clostridium butyricum*, *Enterococcus faecium*, *Lactobacillus acidophilus*, *Lactobacillus casei*, *Lactobacillus fermentum*, *Lactobacillus delbrueckii*, *Lactobacillus helveticus*, *Lactobacillus paracasei*, *Lactobacillus pentosus*, *Lactobacillus plantarum*, *Lactobacillus salivarius*, *Lactococcus lactis*, *Pediococcus pentosaceus*, *Propionibacterium acidopropionici* and *Streptococcus thermophilus* reduce the potency of Caco-2, HT-29, SW1116, HCT116, SW480, DLD-1 and LoVo colon carcinoma cells (75), *Lactobacillus acidophilus* SNUL strains, *Lactobacillus casei* YIT9029 and *Bifidobacterium longum* HY8001 suppress proliferation of human colorectal carcinoma SNUC2A and gastric carcinoma SNU1 cells and *Bacillus polyfermenticus* inhibits colony formation of human colon epithelial cells NMC460. These studies and those revealing immunomodulatory effects of probiotics on cytotoxic T lymphocytes and NK cells indicate their suitability for inclusion in adjuvant therapy, alongside chemotherapy and immunotherapy, in the treatment of some cancers (80).

Synbiotics are combinations between probiotic microorganisms and specific prebiotic compounds, which have the advantage of their simultaneous administration and promote the survival, growth (81) and rapid colonization of probiotics in the gut in a short time, restoring balance and inducing eubiosis state. Synbiotic containing the probiotic *Lactobacillus gasseri* 505 and prebiotic consisting of *Maclura tricuspidata* Carrière (synonym *Cudrania tricuspidata* Bureau) leaf extract in fermented milk shows protection against azoxymethane/dextran sodium sulfate-induced colitis and associated colon carcinoma. It suppresses the incidence of colonic tumors and reduces colonic mucosal lesion formation by inhibiting the secretion of the pro-inflammatory cytokines TNF-α, IFN-γ, IL1-β and IL-6, the inflammation-associated enzymes iNOS and COX2 and the anti-apoptotic factors Bcl2 and BclxL, stimulating the expression of the anti-inflammatory cytokines IL4 and IL10 and the pro-apoptotic factors Tp53, p21 and Bax. At the same time, the synbiotic combination inhibited the growth of bacteria of the genus *Staphylococcus* and maintained the growth of bacteria of the genera *Lactobacillus*, *Bifidobacterium* and *Akkermansia*, which produce short-chain fatty acids and have antitumor effects, suggesting that synbiotic administration may be a promising adjuvant therapy for colorectal carcinoma (82).

**Figure 2:**
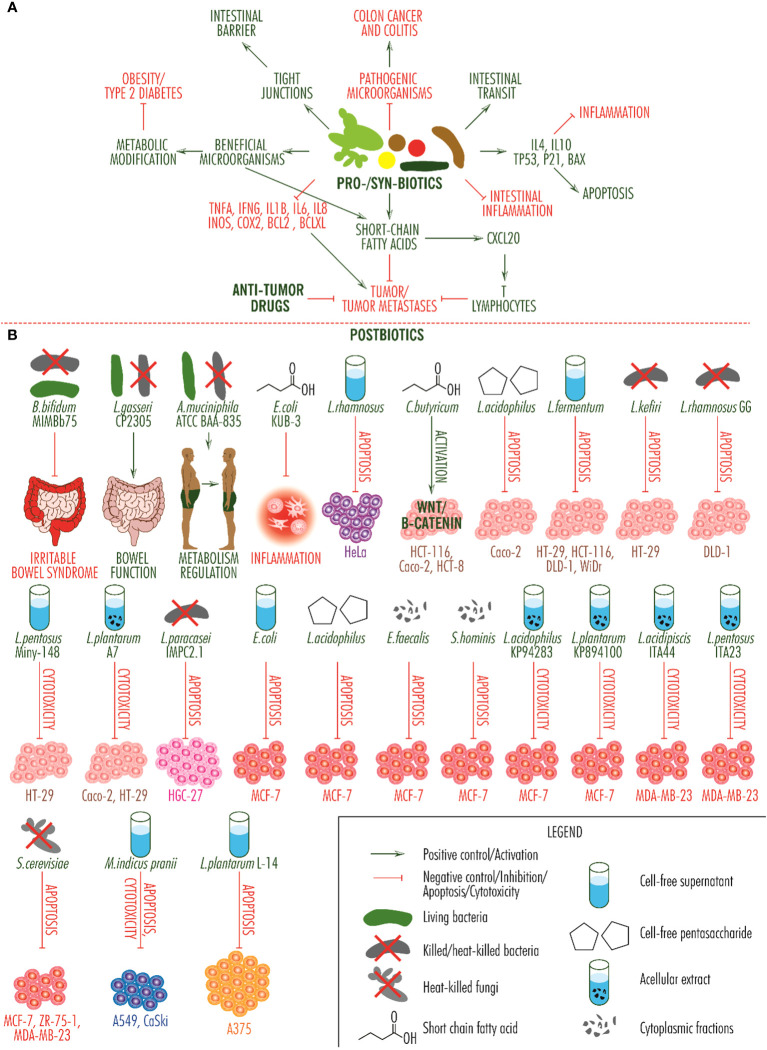
Activity of probiotics, synbiotics and postbiotics. **(A)**. Integrative scheme of probiotic and synbiotic activity. They inhibit the growth of pathogenic microorganisms which cause colitis and colon cancer, regulate intestinal transit, determine the secretion of anti-inflammatory and pro-apoptotic molecules, reduce intestinal inflammation, produce short-chain fatty acids with anti-tumor effects and show a synergistic effect with anti-tumor drugs, inhibit the secretion of pro-tumor molecules (TNF alpha, IFN gamma, IL1beta, IL6, IL8, INOS, COX2, BCL2, BCLXL), contribute to the multiplication of beneficial microorganisms, which improve the metabolism of obese people and those with type 2 diabetes, and contribute to restoring the barrier function of the intestine by restoring tight junctions. **(B)**. Activity of postbiotic products. The effects of relieving symptoms of irritable bowel syndrome, improving intestinal function and regulating the metabolism of obese and overweight people, are present at a systemic level, the fourth, relating to the reduction of inflammation, is produced at a local level and the others are observed *in vitro*, on different cell lines. Unless one postbiotic that induces activation of the Wnt/beta-catenin signaling pathway (this is a signaling pathway with a dual role and which can inhibit tumor development), all other arrows indicate tumor suppressive activity, either through apoptosis, cytotoxicity or both effects. Each cell type category is marked by a specific color: with purple, cervical cancer cell line HeLa; with pink-brown, colorectal cancer cell lines HCT-116, Caco-2, HCT-8, HT-29, DLD-1 and WiDr; with magenta, gastric cancer cell line HGC-27; with red, breast cancer cell lines MCF-7, ZR-75-1 and MDA-MB-23; with blue, long carcinoma cell lines A549 and CaSki; and with orange, skin cancer cell line A375.

Postbiotics are molecules, cell fragments or whole cells derived from microbial cells after they have been killed or inactivated, that produce beneficial health effects. Thus, in viable or inactivated form, *Bifidobacterium bifidum* MIMBb75 reduces the effects of irritable bowel syndrome, *Lactobacillus gasseri* CP2305 in viable form has the same effect and in inactivated form regulates bowel function, and *Akkermansia muciniphila* ATCC BAA-835 in viable or inactivated form improves the metabolism of overweight or obese people (83) and can be used in the prevention of certain types of tumors. On tumor cell lines, postbiotics produce some effects that recommend them as adjuvants in the treatment of some neoplasia. In cervical cancer, acellular *Lactobacillus rhamnosus* supernatant induces apoptosis of HeLa cells; in colorectal cancer, short chain fatty acid from *Clostridium butyricum* activates the Wnt/β-catenin signaling pathway in HCT-116, Caco-2 and HCT-8 cells, cell-free pentasaccharide from *Lactobacillus acidophilus* induces apoptosis of Caco-2 cells, cell-free supernatant from *Lactobacillus fermentum* induces apoptosis of HT-29, HCT-116, DLD-1 and WiDr cells, heat-killed *Lactobacillus kefiri* is apoptotic for HT-29 cell line, *Lactobacillus paracasei* IMPC2.1 and heat-killed *Lactobacillus rhamnosus GG* induce apoptosis of DLD-1 cells, cell-free supernatant from *Lactobacillus pentosus* Miny-148 activates cell-mediated cytotoxicity against HT-29 cells, and heat-killed acellular extract of *Lactobacillus plantarum* A7 activates cell-mediated cytotoxicity against Caco-2 and HT-29 cells; in gastric cancer, heat-killed *Lactobacillus paracasei* IMPC2.1 induces apoptosis of HGC-27 cells; in breast cancer, cell-free supernatant of *Escherichia coli*, cell-free pentasaccharide from *Lactobacillus acidophilus* and heat-killed, cytoplasmic fractions of *Enterococcus faecalis* and *Staphylococcus hominis* induce apoptosis of MCF-7 cells, short chain fatty acid from *Escherichia coli* KUB-36 having anti-inflammatory effect, acellular extracts from heat-killed *Lactobacillus acidophilus* KP94283 and *Lactobacillus plantarum* KP894100 produce cell-induced cytotoxicity on the MCF-7 line, acellular extract of *Lactobacillus acidipiscis* ITA44 and *Lactobacillus pentosus* ITA23 produce cell-induced cytotoxicity on the MDA-MB-23 line, and heat-killed *Saccharomyces cerevisiae* induce apoptosis of MCF-7, ZR-75-1 and MDA-MB-23 cells; in lung carcinoma, cell-free supernatant of heat-killed *Mycobacterium indicus pranii* induces apoptosis and cell cytotoxicity of A549 and CaSki cells, and on the A375 skin cancer cell line, cell-free extract of *Lactobacillus plantarum* L-14 induces apoptosis. *In vivo* studies indicate apoptotic or immune-stimulating activity on colorectal, breast and pancreatic cancers (84) (**Figure 2**).

## 4 The role of the microbiota in cancer therapy

Cancer chemotherapy, referring to the use of traditional cytotoxic chemotherapeutic agents, has been shown to be impacted by the gut microbiome in murine models, particularly with therapies involving cyclophosphamide (CTX) and oxaliplatin (85). Cyclophosphamide can alter the composition of the gut microbiota, leading to the translocation of certain gram-positive bacteria into secondary lymphoid organs triggering the production of “pathogenic” T helper 17 (pTh17) cells while enhancing the response of memory T helper 1 (Th1) cells, thereby bolstering the host immune system (85).

The negative effects of microbiota on chemotherapy are of three types: metabolization and reduced efficacy, increased toxicity with adverse effects, and development of resistance to treatment. For example, the bacterial enzyme uridine phosphorylase (homologue of human uridine phosphorylase) is involved in modulating the activity of capecitabine metabolites. Uridine phosphorylase has a dual role in cancer and is secreted by tumor cells in marginally invasive regions of tumors, especially in those in which TP53 (negative regulator of uridine phosphorylase) is inactivated, and in macrophages in tumor stroma, by a mechanism that includes TNFA and NF-kB, but also by some bacteria, such as *Escherichia coli* and *Parabacteroides distasonis* (86). Gemcitabine, a cytidine analogue used in the first-line treatment of solid tumors of the bladder, pancreas, ovary and breast including pancreatic ductal adenocarcinoma, suppresses cell cycle progression from G1 to S phase and kills cells with active DNA synthesis during S phase (87). In colon carcinoma models, gemcitabine (2’,2’-difluoro-2’-deoxycytidine) is converted to 2’,2’-difluorodeoxycytidine, an inactive metabolite, by an isoform of bacterial cytidine deaminase, produced by *Mycoplasma hyorhinis*, other intratumoral Gamma-proteobacteria and the oral pathogenic bacteria *Aggregatibacter actinomycetemcomitans* and *Porphyromonas gingivalis*, which by this mechanism reduce its efficacy by 10 to 60-fold and induce tumor cell resistance to this drug (88).

Present in the oral cavity, where it causes periodontal disease, *Fusobacterium nucleatum* induces overexpression of TLR4 and BIRC3 (Baculoviral IAP Repeat Containing 3), activates the TLR4-MYD88d and ULK1/ATG7 autophagy network signaling pathways, and genomic loss of miR-18a* and miR-4802, favoring the acquisition of resistance to 5-fluorouracil treatment in people with colorectal cancer (89). Treatment with 5-fluorouracil produces gut dysbiosis, characterized by overgrowth of *Lachnospiracea*_NK4 A136, *Bacteroides*, *Odoribacter*, *Mucispirillum* and *Blautia* and reduction of *Coriobacteria* and *Deltaproteobacteria* taxa (90), with induction of side effects, and oral dysbiosis, favoring depletion in *Streptococcus*, *Actinomyces* and *Veillonella* and multiplication of *Prevotella oris* and *Fusobacterium nucleatum*, which favor the occurrence of oral mucositis, as a side effect of chemotherapy (91).

Gut microbiota and fecal microbiota transplantation may play an important role in the limitation of oxaliplatin adverse effects, including peripheral neurotoxicity (numbness of extremities, hyperalgesia), which are not present in laboratory animals pretreated with a mixture of antibiotics (92).

Doxorubicin is a drug of the anthracycline class, naturally produced by the actinobacterium *Streptomyces peucetius* var. *casieus* and used in the treatment of several types of tumors, which it inhibits by inducing damage to genetic material. However, taken in high doses, it has side effects on the heart. Under anaerobic conditions, the electron transport chain NADH dehydrogenase enzyme produced by *Streptomyces* WAC04685 inactivates it by deglycosylation (93), and a molybdopterin-dependent enzyme from *Escherichia coli* BW25113 and *Klebsiella pneumoniae* degrades it, reducing its efficiency. *Raoultella planticola* has an antagonistic effect on them, restoring the therapeutic efficacy of doxorubicin (94).

Irinotecan/CPT-11 is a drug used together with 5-fluorouracil in the treatment of colon cancer. Administered in glucuronic acid conjugated form, irinotecan is deconjugated by the enzyme β-glucuronidase secreted by *Escherichia coli*, favoring the manifestation of drug toxicity, reduced antitumor efficacy and the onset of severe diarrhea, one of its main side effects (95). Administration of a low dose of amoxapine, an antidepressant drug that inhibits β-glucuronidase without affecting the gut microbiota, together with irinotecan prevents diarrhea and contributes to the manifestation of the antitumor activity of the latter (96).

Preclinical studies have demonstrated that depletion of the gut microbiota can sustain the survival of transferred T cells in a cervical cancer model treated with adoptive T cell therapy (ACT), reliant on systemic CD8α + dendritic cells (DCs) and interleukin-12 (IL12) (97).

Specific members of the gut microbiome, namely *Enterococcus hirae* and *B. intestinihominis*, can impact the clinical efficacy of CTX in cancer treatment by reducing regulatory T cells and enhancing the immune response of MHC class I-restricted cytotoxic T cells (CTLs) against the tumor, thereby modifying the tumor microenvironment (56).

Another study conducted on the FOLFOX chemotherapy regimen (comprising 5-FU, leucovorin calcium, and oxaliplatin) in a colorectal cancer model revealed that certain microbiome compositions can trigger the activation of nuclear transcription factor-κB (NF-κB) and enhance the production of interleukin-6 (IL-6) and tumor necrosis factor (TNF). This cascade promotes inflammation and leads to mucosal damage (98). However, the probiotic *Lactobacillus rhamnosus* has been shown to mitigate chemotherapy-induced mucositis by modulating the proinflammatory response and suppressing intrinsic apoptosis in intestinal injury.

The intestinal microbiota can influence the adverse drug reactions associated with irinotecan-based chemotherapy by reactivating the metabolite of SN-38 glucuronide (99). In the study by Guthrie et al., the abundance of Faecalibacterium prausnitzii and specific *Bacteroides* species varied significantly among cohorts stratified based on glucuronide metabolism. Inhibiting microbial β-glucuronidases could potentially alleviate severe side effects induced by irinotecan, such as severe diarrhea (99).

The advent of immune checkpoint inhibitors (ICIs) in cancer treatment, encompassing monoclonal antibodies directed against programmed death receptor (PD-1), programmed death receptor ligand (PD-L1), and cytotoxic T lymphocyte-associated protein 4 (CTLA-4) receptor, has heralded a transformative era in cancer therapy, drastically altering the prognosis of numerous malignancies. These agents are extensively employed in the management of advanced-stage cancer but the emergence of primary and secondary resistance to cancer treatment poses a significant challenge and can profoundly impact patient outcomes (100). Recently, emerging evidence has underscored the profound influence of the gut microbiota on tumor response to ICIs, as demonstrated in both clinical cohorts and preclinical mouse models.

Several studies have identified Bacteroidetes as a biomarker indicating non-responsiveness to immune checkpoint inhibitors in patients with metastatic melanoma (MM) (101, 102).(Its presence may reduce response rates and dampen systemic and antitumor immunity, potentially lowering the risk of local inflammation such as ICI-induced colitis. However, specific strains of Bacteroidetes, such as *Bacteroides thetaiotamicron* and *B. caccae*, have been linked to an effective therapeutic response (103).

Radiation therapy can also trigger apoptosis of intestinal cells and disrupt the composition of the gut microbiome, resulting in intestinal inflammation, which can manifest as symptoms like diarrhea and fatigue (104). Side effects such as fatigue, nausea, vomiting, and diarrhea associated with radiation therapy may be alleviated by probiotics like Lachnospiraceae and Enterococcaceae, indicating a potential for reducing radiation-induced damage through modulation of the gut microbiome (105). Notably, findings from a randomized clinical trial revealed that combining probiotics with radiation therapy in patients with nasopharyngeal carcinoma undergoing concurrent radiochemotherapy could significantly enhance host immunity and alleviate oral mucositis (OM) associated with radiochemotherapy by altering the gut microbiota (106).

## 5 Current challenges - the intratumor microbiota

Current research is focused on locally resident microbiota and intratumor microbiota. Locally resident microbiota, particularly those inhabiting the gastrointestinal tract and other parts of the digestive system, have been identified as closely linked to the carcinogenesis of their respective organs. Previous studies have demonstrated associations between susceptibility to various cancers, such as oral squamous cell carcinoma (OSCC), esophageal cancer, gastric cancer, gastric diffuse large B cell lymphoma (DLBCL), colorectal cancer (CRC), gastric mucosa-associated lymphoid tissue lymphoma (MALT), hepatocellular carcinoma (HCC), pancreatic cancer, gallbladder cancer, lung cancer, breast cancer, and prostate cancer, and locally resident microbiota (107).

However, the relationship between locally resident microbiota and the efficacy of therapy remains under investigation. Notably, for patients with colorectal cancer (CRC), the gut microbiome also constitutes the locally resident microbiota at the tumor site. The significance of the intratumor microbiome in gastrointestinal cancer also plays a pivotal role in determining the effectiveness of cancer treatment. In a study focusing on murine colon cancer, Geller discovered that intratumor Gammaproteobacteria can impact gemcitabine metabolism, leading to resistance against the drug (88).

Eliminating bacteria residing within pancreatic cancer was associated with the immune response to PDAC. Modifying the tumor microenvironment to hinder the infiltration of myeloid-derived suppressor cells (MDSCs) promoted the differentiation of M1 macrophages which, in turn, facilitated the differentiation of Th1 cells and augmented the population of activated CD4+ and CD8+ T cells (108). The elimination of bacteria could potentially alter the response of PDAC patients to ICIs by increasing the expression of PD-1. Regarding the precise mechanism of immune reprogramming, the microbiome associated with PDAC can activate specific Toll-like receptors in monocytic cells, leading to the induction of a tolerogenic immune program that suppresses both innate and adaptive immunity (108).

A recent study compared bronchial brushing samples from 24 lung cancer patients and 18 healthy controls to investigate the airway microbiome. Samples from patients included unilateral lobar tumor sites and paired samples from both the cancerous and noncancerous sites. The findings revealed differences in the microbiota profiles between cancerous sites of lung cancer patients and healthy controls, with lower microbial diversity observed in cancerous sites and healthy controls compared to noncancerous sites. The tumor tissue exhibited higher abundance of Streptococcus and Neisseria, whereas *Staphylococcus* and *Dialister* were more prevalent in normal tissue. Additionally, there was a gradual shift in microbiota abundance from normal tissue to noncancerous site tissue to cancerous tissue in lung cancer patients. These findings suggest that the lung microbiota can significantly influence the tumor microenvironment, impacting cancer progression and patient prognosis beyond just the cancerous site (109).

The microbiota within tumors could serve as a source of non-self antigens, capable of being recognized by T cells and influencing responses to therapy. For instance, a recent analysis of metastases from 17 melanoma patients revealed a variety of peptides binding to MHC class I and MHC class II, originating from 41 bacterial species (110). Moreover, T cells specific to the microbiota might contribute to certain immune checkpoint blockade (ICB)-induced immune-related adverse events. An investigation demonstrated that ICB-induced dermatitis might arise from T cells targeting epithelial cells presenting antigens from skin commensals. In mice, the introduction of *Staphylococcus epidermidis* to the skin during anti-CTLA4 treatment prompted epithelial inflammation driven by S. epidermidis-specific T cells producing IL-17 (110).

Intratumoral microbes have the ability to evade immune responses, impacting tumor development by fostering an immunosuppressive environment and deactivating immune cells. For example, *F. nucleatum* influences the tumor immune landscape by specifically attracting tumor-infiltrating myeloid cells such as CD11b+ myeloid cells, MDSCs, tumor-associated macrophages, classical myeloid DCs, and CD103+ regulatory DCs, thereby enhancing tumorigenesis (111). Moreover*, F. nucleatum* has the ability to attach to and trigger the T Cell immunoreceptor with immunoglobulin and immunoreceptor tyrosine-based inhibitory motif domains (TIGIT), as well as the carcinoembryonic antigen cell adhesion molecule 1 (CEACAM1) receptors present on human NK cells and other lymphocytes thereby suppressing the function of anti-tumor immune cells in colorectal cancer (CRC) (112).

In a study conducted on mice, it was found that Pasteurella showed a positive correlation with cytotoxic CD8+ tumor-infiltrating lymphocytes (TILs) and a negative correlation with M2-like macrophages. Conversely, Coriobacteriaceae exhibited a positive association with M2-like macrophages and a negative association with CD8+ cells. These various immune reactions play a significant role in the onset and progression of lung tumors (113).

While the intratumor microbiome can diminish the potency of chemotherapeutic agents, suppress the expression of major histocompatibility complex (MHC) class I, and elevate MDSC numbers, it can also prompt alternative immune checkpoints and impede lymphocyte clonal expansion (114). Conversely, it has the capacity to directly engage the innate immune system, generate anti-inflammatory cytokines, and heighten the expression of targetable checkpoint molecules, potentially bolstering cancer immunity (114). This dual impact on the immune microenvironment highlights the complexity of the tumor microbiome and warrants further investigation across diverse cancer types.

In essence, the influence of locally resident microbiota or intratumor microbiota on cancer therapy remains inadequately explored (115). Although the roles of certain locally resident or intratumor bacteria have been confirmed in select cases, the majority of studies have focused on gut-related scenarios. Evidence regarding the interaction between cancer therapy efficacy and locally resident microbiota in other areas of the digestive tract is lacking. Nevertheless, there are indications linking locally resident microbiota with local inflammation and cancer progression, suggesting that modifying the microbiome could enhance patient prognosis.

## 6 Conclusions

Microorganisms are an important component of the human organism, forming a dense ‘organ’ with a specific signature of each individual and with a role in nutrition, in regulating epithelial development and in training innate immunity. The body includes numerous non-sterile sites, each developing a characteristic microbiota. Under conditions of eubiosis, it develops relationships of commensalism and symbiosis with the organism, contributing to an important extent to health and protecting it from some diseases, but under conditions of dysbiosis, microorganisms are involved in several pathologies, including cancer. Many recent papers highlight the roles of human microbiota in different aspects of carcinogenesis, from cancer susceptibility and progression to anticancer therapy response, but the elucidation of the mechanistic implications is still in its infancy. Microbiota can modulate anticancer therapy efficacy and toxicity, and also influence resistance to anti-cancer drugs due to its ability to metabolize drugs and xenobiotics and to modulate host inflammation and immune responses. Microbiome-based therapeutic interventions may be able to correct dysbiosis, maximize the response to anticancer treatments and prevent carriage of antimicrobial-resistant pathogens. A better knowledge of gut microbiota roles in cancer will enable us to develop novel microbiome derived biomarkers, anticancer treatment strategies and subsequently improve the cancer patients’ outcome.

## Author contributions

MC: Writing – original draft. MCC: Writing – original draft, Conceptualization, Formal analysis, Funding acquisition. GM: Writing – original draft. NC: Writing – review & editing. LB: Writing – review & editing. CB: Writing – review & editing. ST: Writing – review & editing, Formal analysis. MM: Writing – review & editing. RF: Writing – original draft. SG-M: Writing – review & editing. GP: Writing – original draft, Writing – review & editing.
